# Nine-Gene Prognostic Signature Related to Gut Microflora for Predicting the Survival in Gastric Cancer Patients

**DOI:** 10.5152/tjg.2024.23063

**Published:** 2024-02-01

**Authors:** Qing Yue, Wei Han, Zi-Ling Liu

**Affiliations:** 1 Department of Oncology, Cancer Center, The First Hospital of Jilin University, Changchun, Jilin, China; 2 Department of Hepatobiliary and Pancreatic Surgery, General Surgery, The First Hospital of Jilin University, Changchun, Jilin, China

**Keywords:** Gastric cancer, gut microflora, nomogram, prognosis

## Abstract

**Background/Aims:**

The purpose of this study is to screen the feature genes related to gut microflora and explore the role of the genes in predicting the prognosis of patients with gastric cancer.

**Materials and Methods:**

We downloaded the gene profile of gastric cancer from the University of California Santa Cruz, the gut microflora related to gastric cancer from The Cancer Microbiome Atlas. The GSE62254 dataset was downloaded from National Center for Biotechnology Information Gene Expression Omnibus as a validation dataset. A correlation network between differentially expressed genes and gut microflora was constructed using Cytoscape. The optimized prognostic differentially expressed genes were identified through least absolute shrinkage and selection operator (LASSO) algorithm and univariate Cox regression analysis. The risk score model was established and then measured via Kaplan–Meier and area under the curve. Finally, the nomogram model was constructed according to the independent clinical factors, which was evaluated using C-index.

**Results:**

A total of 754 differentially expressed genes and 8 gut microflora were screened, based on which we successfully constructed the correlation network. We obtained 9 optimized prognostic differentially expressed genes, including *HSD17B3*, *GNG7*, *CHAD*, *ARHGAP8*, *NOX1*, *YY2*, *GOLGA8A*, *DNASE1L3*, and *ABCA8*. Moreover, Kaplan–Meier curves indicated the risk score model correctly predicted the prognosis of gastric cancer in both University of California Santa Cruz and GSE62254 dataset (area under the curve >0.8; area under the curve >0.7). Finally, we constructed the nomogram, in which the C index of 1, 3, and 5 years was 0.824, 0.772, and 0.735 representing that the nomogram was consistent with the actual situation.

**Conclusions:**

These results indicate the 9 differentially expressed genes related to gut microflora might predict the survival time of patients with gastric cancer. Both risk signature and nomogram could effectively predict the prognosis for patients with gastric cancer.

**Main Points**

- We screened 9 differentially expressed genes related to gut microflora that might be associated with prognosis of gastric cancer (GC).
- The risk score and nomogram model could effectively predict the prognosis for GC.

## Introduction

Gastric cancer (GC) is an incurable malignant tumor worldwide, which led to about 800 000 deaths based on GLOBOCAN 2020 estimates and is regarded as the fourth leading cause of cancer deaths.^1^ Surgical treatment is still considered as the first line of therapy for patients with GC.^2,3^ Nevertheless, effective therapeutic strategies for GC patients include surgery, neoadjuvant, therapy, radiotherapy, and chemotherapy. However, statistical evidence has revealed that GC patients remain to have a low 5 overall survival rate.^4^ Besides, due to the high recurrence rate of GC patients treated with surgery or other treatment methods, patients have a poor prognosis.^5,6^ Therefore, reducing the incidence of GC remains the key to reducing mortality.

Studies have confirmed that gut microflora participates in the body’s nutrition, metabolism, and immunity.^7,8^ The changes in gut microflora can also lead to the occurrence and development of inflammation.^9^ Moreover, previous study has reported that the occurrence and progression of GC are involved in various viral, parasitic, or bacterial infections.^10^ It is reported that due to the extreme acidity of the stomach, its microbial abundance is the lowest.^11^ Microbial species in the gastrointestinal tract constitute the microbiota, which refers to the microbial ecological community found in a specific environment. The interaction of microbiota with different types of host cells can regulate physiological functions and organ microenvironment.^12^ The process of GC has a close association with the changes of cell pathophysiology and microbial characteristics.^13,14^ Besides, gut microflora in GC has a significant effect on the clinical outcomes of chemotherapy, radiotherapy, and immunotherapy, indicating that gut microflora may be the novel targets for improving antitumor therapy. With the development of modern molecular biology technology, the deepening of metagenomics research, and the progress of metabolomics, more attention has been paid to the study of gut microbiota and its metabolism. The relationship between GC and gut microflora has attracted more attention. There are many reports on the relationship between human gut microflora and GC. 

Most reports focused on the different genera of bacteria in the occurrence and development of tumors. Ai et al^15^ indicated that bacteria (such as Helicobacter, Streptomonospora, and Acinetobacter) may be involved in tumor progression as potential characteristic genera. Besides, Li et al^16^ identified novel and consistent microbial patterns in gastric carcinogenesis. However, there are few reports on the relationship between microbiota and prognosis in GC. Therefore, based on the clinical prognostic information and gene expression profiles of the cancer genome atlas (TCGA) samples and combined with the database The Cancer Microbiome Atlas (TCMA) of gut microbiota, we aimed to search for the microorganisms related to the prognosis of gut microbiota in GC. In addition, we characterized the clinical and functional characteristics of different phenotypic samples based on transcriptome data and then explored key genes related to gut microbiota in GC.

## Materials and Methods

### Data Source and Preprocessing

The gene expression level (normalized log^(FPKM+1,2)^ expression value) of GC was obtained from the University of California Santa Cruz (UCSC) Xena (https://xenabrowser.net/datapages/). Subsequently, we obtained 407 samples based on the platform of Illumina HiSeq 2000 RNA Sequencing. Meanwhile, we obtained the GC-related microflora data including 166 samples from The Cancer Microbiome Atlas (TCMA, https://tcma.pratt.duke.edu/). ^17^ We retained the samples that were detected in both UCSC and TCMA databases. According to the clinical information of samples, we obtained 91 GC samples and 9 control samples with clinical prognosis information that were regarded as the training dataset.

Besides, we downloaded the GSE6225 including 300 samples with clinical prognosis information from National Center for Biotechnology Information Gene Expression Omnibus (NCBI GEO, https://www.ncbi.nlm.nih.gov/geo/) ^18^ on the basis of the platform of GP570 Affymetrix Human Genome U133 Plus 2.0 Array. This dataset was considered as a validation dataset to establish a survival prognostic model.

### Screening of Differentially Distributed Microflora and Expressed Genes

According to the GC-related microflora data in the TCMA database, the *t*-test of R 3.6.1 (http://127.0.0.1:15190/library/stats/html/t.test.html) was applied for comparing the microflora distribution between GC tumor and normal samples. The cutoff was set as *P* < .05.

Then, the limma package (Version 3.34.7, https://bioconductor.org/packages/release/bioc/html/limma.html) ^19^ in R 3.6.1 was carried out to screen the differentially expressed genes (DEGs) from GC tumor and normal samples of GC-related gene expression profiling data in UCSC. The threshold was set to false discovery rate (FDR)<0.05且|log2FC|>1. The expression of the screened DEGs was exhibited using heatmap (https://cran.r-project.org/web/packages/pheatmap/index.html) ^20^ that was constructed using bidirectional hierarchical clustering analysis on the basis of centered Pearson correlation algorithm.^21^

### Screening of Differentially Expressed Genes Significantly Related to Gut Microflora Distribution

The cor function in R3.6.1 (http://77.66.12.57/R-help/cor.test.html) was performed to calculate the Pearson correlation coefficient (PCC) between the screened DEGs and species of gut microbiota with significantly different distributions. The cutoff was set for *P* < .05 and |PCC| > 0.3. Subsequently, the related network was constructed based on the significantly correlated pairs, which were visualized through Cytoscape (Version 3.6.1, https://cytoscape.org/). ^22^ After that, the Gene Ontology Biology Process (GO-BP) and Kyoto Encyclopedia of Genes and Genomes (KEGG) based on DAVID (Version 6.8, https://david.ncifcrf.gov/) ^23,24^ were carried out to analyze the function and pathways on the DEGs related to gut microflora with the threshold of *P* < .05.

### Construction of Survival Prognosis Model

According to the clinical information of samples including survival prognosis, the univariate Cox regression analysis (Version 2.41-1, http://bioconductor.org/packages/survivalr/) of the survival package in R3.6.1 ^25^ was carried out to identify the DEGs correlated with prognosis. The value of log-rank *P* < .05 was regarded as significant. Then, we carried out regression analysis via the LASSO algorithm of the lars package in R3.6.1 (Version 1.2, https://cran.r-project.org/web/packages/lars/index.html) ^26^ on DEG-related prognosis to screen the optimized DEG combinations.

According to the LASSO prognostic coefficient and expression level of the DEG combinations, we established the risk score (RS) model using the following formula: RS = ∑ Coef_DEGs_ × Exp_DEGs_. In this formula, Coef_DEGs_ indicate the prognostic coefficients of DEGs and Exp_DEGs_ represent the expression of target DEGs.

To evaluate the efficacy of the RS prognosis model, we first calculated the RS value in the UCSC training dataset. Then, we separated the GC samples into low- and high-risk groups using the RS median value. The Kaplan–Meier (KM) analysis of the survival package in R3.6.1 (Version 2.41-1)^25^ was carried out to assess the correlation between grouping situation of high- or low-risk groups and actual information on patients’ prognosis. Meanwhile, the KM curves of the GSE62254 validation dataset were obtained using the same method as the UCSC training dataset.

### Analysis of Clinical Factors

To analyze the clinical information from high-risk group to low-risk group, Fisher’s exact test in R3.6.1 was conducted for the UCSC GC tumor samples. Then, univariate and multivariate Cox regression analyses^25^ were performed to obtain the independent survival prognosis clinical factors with the threshold of log-rank *P* < .05. Furthermore, combining with the risk grouping discriminated by the prognostic prediction model, we constructed the 1, 3, and 5 years nomogram based on the screened independent clinical elements via the rms package (https://cran.r-project.org/web/packages/rms/index.html). ^27,28^ After that, the survcomp (Version 1.34.0) in R3.6.1 was used to calculate the C-index coefficient of nomogram.^29^ A C-index above 0.70 indicated a good model.^30,31^

### Correlation Analysis Between the Differentially Expressed Genes and the Distribution of Gut Bacteria

To study the correlation between feature DEGs and the distribution of gut bacteria, we conducted the cor function in R3.6.1 to compute the PCC value between the expression level of the feature DEGs and gut bacteria. The results were shown as the correlation heatmap.

## Results

### Screening of Differentially Distributed Microflora and Expressed Genes

**Figure 1.**
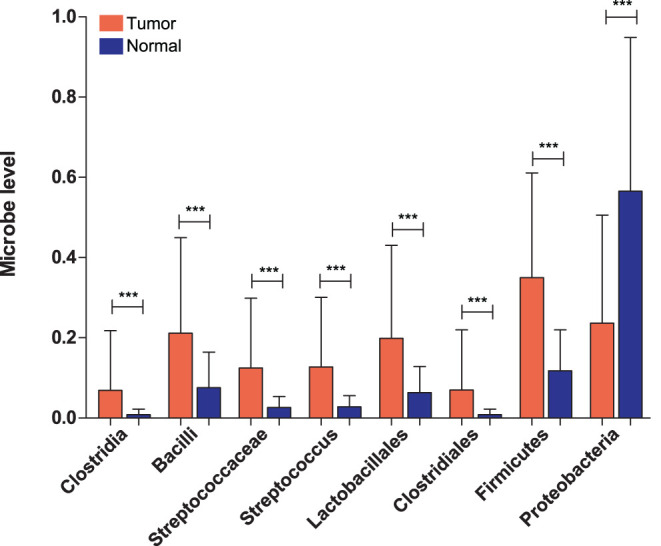
Screening of differentially distributed microflora and expressed genes. Distribution map of gut microbiota with significantly different distributions in gastric cancer tumor and normal control samples. ^***^ *P* < .005.

**Figure 2.**
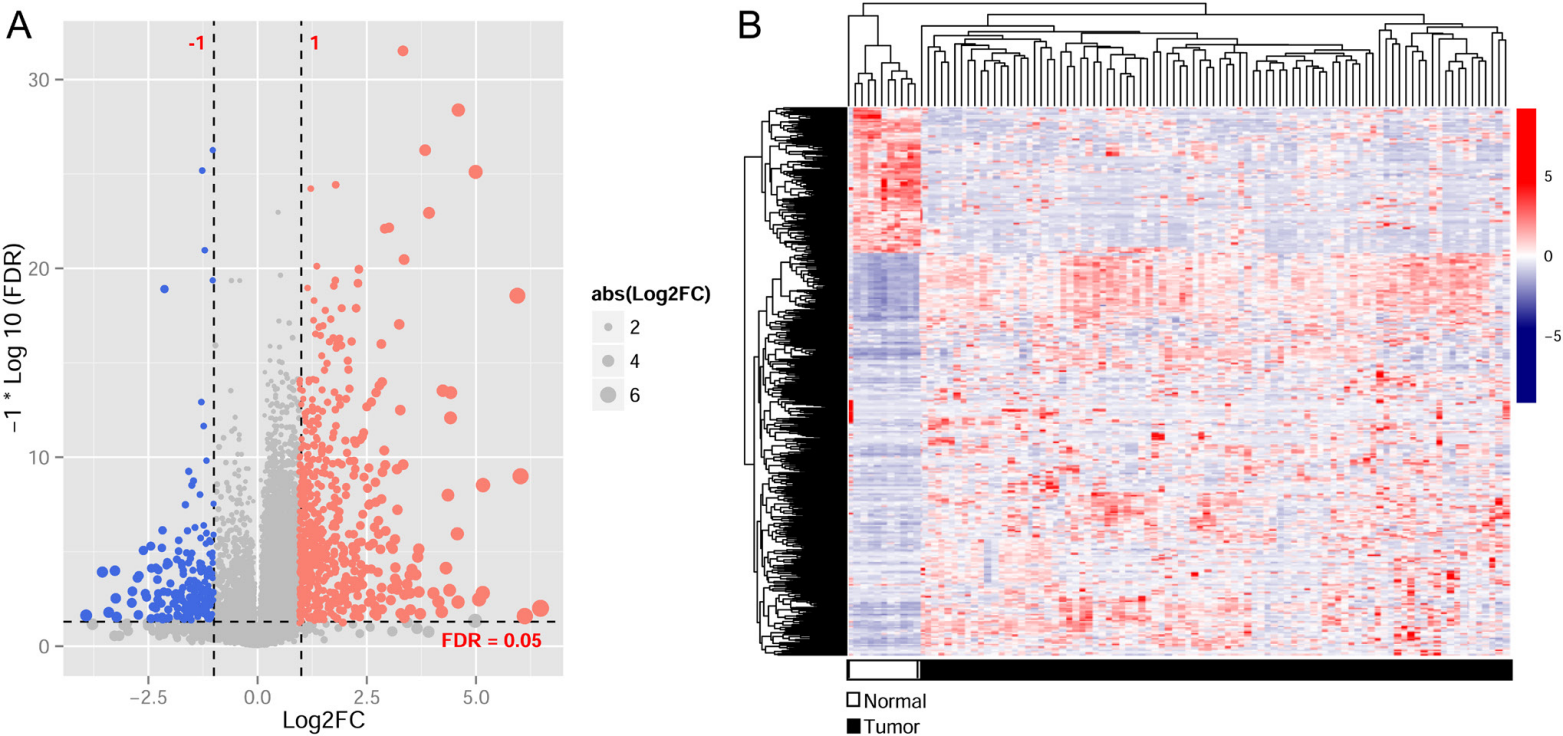
Screening of differentially expressed genes. (A) Volcano plot, red and blue dots indicate significantly differentially up- and downregulated genes, vertical dotted lines indicate FDR < 0.05, and 2 horizontal dotted lines indicate |log2FC|>1. (B) Heatmap showed the expression levels of differentially expressed genes in gastric cancer (GC)and normal control samples. The white and black in the lower sample represent normal and GC tumor samples, respectively.

A total of 91 GC tumor samples were obtained after the comparison between UCSC and TCMA datasets. As shown in Figure 1, 8 gut bacteria were significantly associated with GC using *t*-test, in which the distribution of Clostridia, Bacilli, Streptococcuaceae, Streptococcus, Lactobacillales, Clostridiales, and Firmicutes in tumor was higher than that in normal samples (*P* < .01). However, the distribution of Proteobacteria in GC tumor samples was significantly lower than that in normal control samples (*P* < .01). The types and distribution information of the differentially expressed flora are shown in Table 1. Besides, we obtained 754 DEGs in the UCSC training dataset using the limma method (Figure 2A). The heatmap of the 745 DEGs was shown in Figure 2B, indicating that screened DEGs were consistent in the degree of difference and the direction of dysregulation in the dataset.

### Screening of Differentially Expressed Genes Significantly Related to Gut Microflora Distribution

According to the PCC value between DEGs and 8 types of gut flora, we constructed the correlation network including 332 significantly correlated pairs. This network contained 212 nodes, of which 8 nodes were gut microbiota and 204 were DEG nodes. Among these DEGs, *COL11A1* was correlated with both Proteobacteria and Firmicutes, *KRTAP3-1 *was associated with Clostridia and Clostridiales. Then, the GO and KEGG results indicated that these 204 DEGs were involved in 20 GO BPs, such as GO:0006260~DNA replication, GO:0070268~cornification and GO:0031424~keratinization, and 8 KEGG pathways including hsa00830:Retinol metabolism, hsa03440:Homologous recombination and hsa05150:*Staphylococcus aureus* infection, and so on (Table 2).

### Construction of Survival Prognosis Model

**Figure 3.**
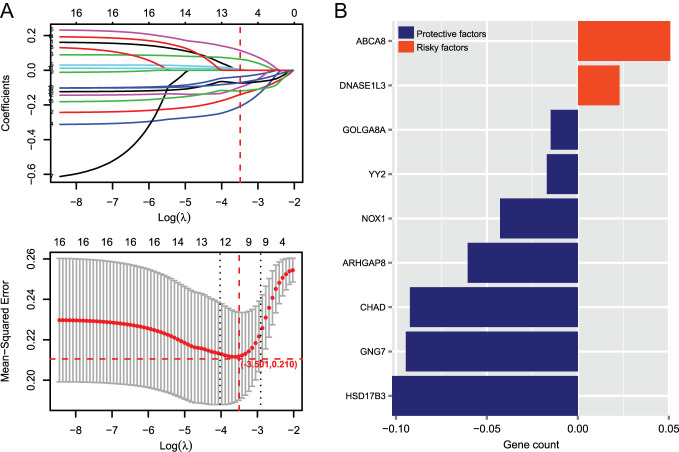
The screening of optimized prognostic genes for gastric cancer. (A) LASSO parameter map, the place where the horizontal and vertical red dotted lines intersect in the figure is the position where the optimal parameter is obtained, and the corresponding gene at this time is the optimal gene combination. (B) Display of the LASSO prognostic coefficients of the optimized differentially expressed genes.

A total of 16 DEGs were related to prognosis based on the 204 DEGs in the correlation network using univariate Cox regression analysis. We then obtained 9 optimized DEGs using the LASSO method. As shown in Figure 3A, when the parameter -log(lambda) is −3.501, the lowest mean-squared error of 0.210 was obtained. At this time, the number of corresponding gene variables is 9, and the 9 genes shown in Figure 3B are the optimal ones, including *HSD17B3*, *GNG7*, *CHAD*, *ARHGAP8*, *NOX1*, *YY2*, *GOLGA8A*, *DNASE1L3*, and *ABCA8*. Furthermore, we conducted the KM survival analysis to study the correlation between the expression of 9 optimized DEGs and patients’ prognosis. Furthermore, we found that high expression of *CHAD*, *GNG7*, *GOLGA8A*, *YY2*, *ARHGAP8*, *HSD17B3*, and *NOX1* was significantly correlated to good survival outcomes. In contrast, *DNASE1L3* and *ABCAB* were highly expressed in samples with GC leading to poor prognosis.

**Figure 4.**
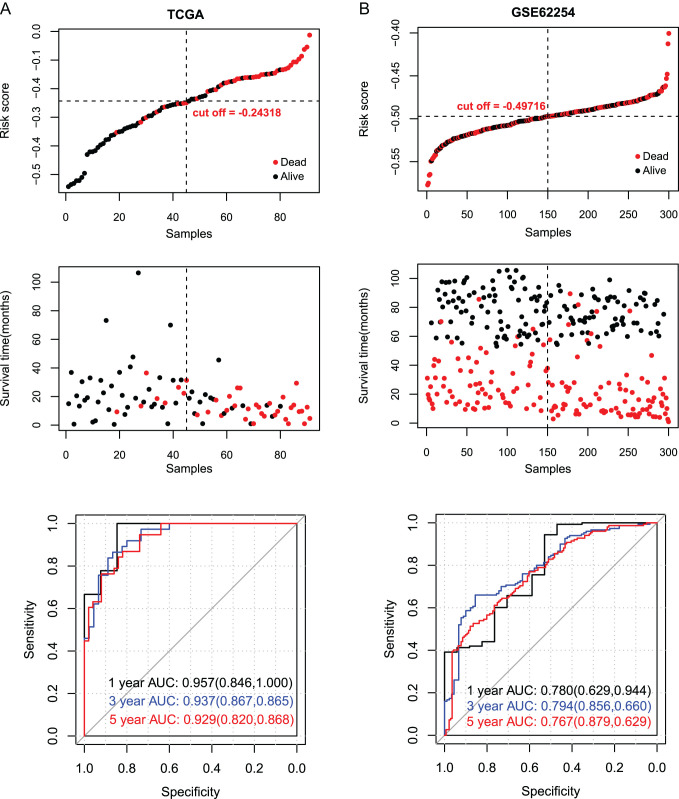
Evaluation and comparison of the efficacy of risk score prediction model. University of California Santa Cruz training set (A), GSE62254 (B) validation set risk score distribution (top panel), survival time status (middle panel), and receiver operating characteristic curve graph based on gene prognostic features (bottom panel), the numbers in parentheses indicate the corresponding receiver operating characteristic curve specificity and sensitivity.

**Figure 5.**
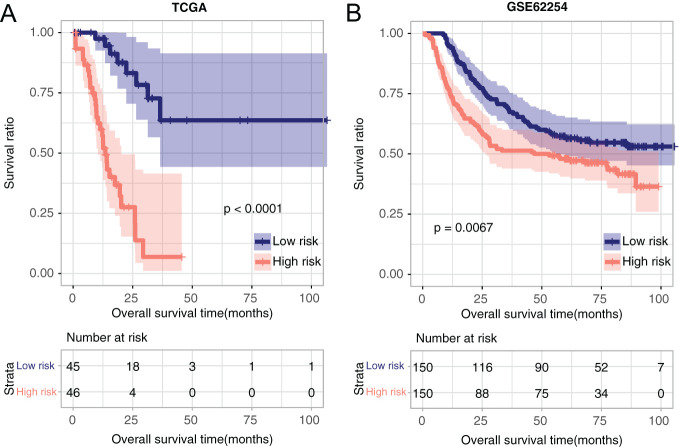
The correlation between grouping and actual prognosis information. (A) University of California Santa Cruz training set; (B) GSE62254. The blue and red curves represent low- and high-risk samples, respectively.

Besides, we divided the samples into high- or low-risk groups according to the RS value. We exhibited the RS distribution and patients’ survival time in Figure 4A. The ROC curves of 1, 3, and 5 years in the UCSC training dataset showed good prediction with the area under the curve (AUC) of 0.957, 0.937, and 0.929. Also, the ROC curves of 1, 3, and 5 years in the GSE62254 validation dataset had good predictive ability with the AUC of 0.780, 0.940, and 0.767, respectively (Figure 4B). After that, to validate the predictive ability of the RS model on the prognosis of GC patients, we constructed the KM curves. The results indicated that there is an obvious relation between the different risk groups based on the RS model and the actual prognosis (Figure 5). In the TCGA dataset, we found that lower risk was related to longer survival (Figure 5A), at the same time, the same results were obtained in the GSE62254 dataset (Figure 5B).

### Analysis of Clinical Factors

**Figure 6.**
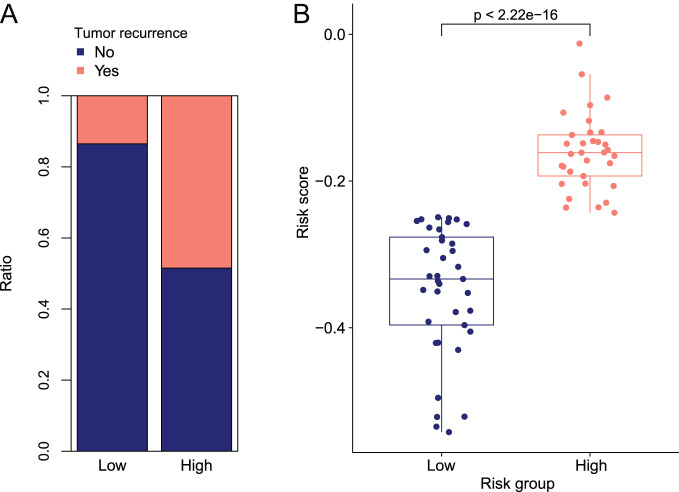
Analysis of clinical information for different risk grouping. (A) The number of different recurrence clinical factors in different risk groups. (B) Differences in the distribution of risk score values in different recurrence clinical groups.

We compared the clinical information between high-risk group and low-risk group using Fisher’s exact test. As indicated in Table 1, the results expressed that there was a significant difference in the distribution of recurrence factors in both high- and low-risk groups (*P* < 1.82E-03). Besides, as shown in Figure 6A, the patients in the low-risk group had less risk of recurrence than that in the high-risk group. And the distribution of RS between different recurrence factor groups was shown in Figure 6B.

**Figure 7.**
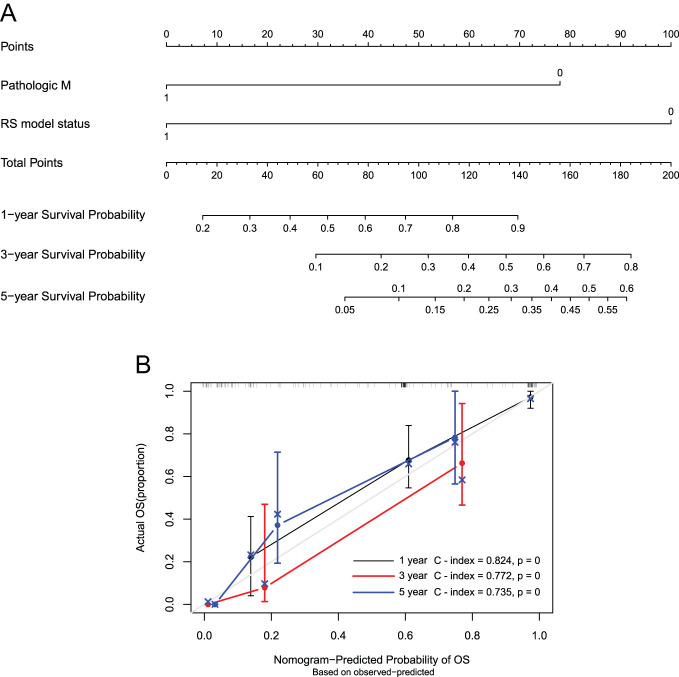
Construction of nomogram survival model for independent prognostic factors. (A) Nomogram plot of the independent prognostic factor column chart survival prediction model. (B) A line chart showing the consistency between 1-year, 3-year, and 5-year survival rate predictions and actual survival rates. The horizontal axis represents the predicted survival rate, while the vertical axis represents the actual survival rate.

We screened 2 independent factors related to prognosis including Pathologic M and RS model (Table 3). After that, to analyze the relation between clinical elements (PathologicM and RS model status) and the patients’ survival prognosis, we constructed the nomogram model shown in Figure 7A. Besides, the C index of 1, 3, and 5 years was 0.824, 0.772, and 0.735, respectively, representing that the nomogram survival model was consistent with the actual situation, indicating that this model exactly predicted the survival time of patients (Figure 7B).

## Discussion

Studies have found that the composition of gut microbiota can not only affect tumor stress but also shape the microbiota for tumor survival and the tumor microenvironment suitable for growth.^32,33^ The TCMA database has greatly aided in the identification of microbial communities and abundances derived from human tissues and organs.^17^ Existing evidence has illustrated the links between gut microbiota and GC.^34,35^ However, few studies have indicated the association between the prognosis of GC patients and gut microbiota. In this study, 9 DEGs related to prognosis were screened that have a significant correlation with gut microbiota. Based on the 9 DEGs, we successfully constructed the RS model that could correctly predict the prognosis of GC patients. 

Recently, 16S rRNA sequencing has helped exploring novel biomarkers related to gut microbiota in GC.^36^ Numerous studies have indicated that novel biomarkers might impact the composition and diversity of stomach microbiome in the procession of GC.^37-39^ In this study, we screened 9 prognosis DEGs related to gut microbiota including *HSD17B3*, *GNG7*, *CHAD*, *ARHGAP8*, *NOX1*, *YY2*, *GOLGA8A*, *DNASE1L3*, and *ABCA8*. Among the 9 DEGs, 2 genes (*ABCA8* and *DNASE1L3*) were the risk factors for GC, the high expression of which might lead to poor survival of GC patients. Previous studies have reported that the 9 DEGs had the prognostic ability for predicting the survival time for GC patients.^40,41^ A risk model based on the 9 DEGs could well predict the prognosis for GC patients with an AUC of 0.957. Furthermore, we found that lower risk scores were related to better long-term survival. 

Moreover, a nomogram consisting of clinical factors and a risk model led to an increase in the predictive accuracy of GC patients’ prognosis.^42^ Li et al^43^ reported that the risk signature alone predicted the long-term survival of GC patients for 1-, 3-, and 5-year survival with an accuracy of 0.644, 0.72, and 0.779, respectively. These results suggested the nomogram predicted the long-term survival of GC patients. Our study obtained similar results. In our study, we successfully constructed the RS model using the 9 DEGs. The 1-, 33, and 5-year AUC value of the RS model in the UCSC dataset was above 0.9, suggesting that the RS model could correctly predict the prognosis of GC. Meanwhile, we also found similar results as in the validation dataset. Besides, we further analyzed the correlation between survival ratio and risk grouping, the results demonstrated that the low-risk group was associated with a good survival ratio. All the findings concluded that the RS model based on 9 genes has an accurate prediction for the prognosis for GC. All these findings indicated that the 9 DEGs were involved in constituting GC prognostic model. Thus, the 9 DGEs might play an important role in predicting the prognosis for GC patients. Further function experiments (vivo and vitro tests) are necessary to be performed for validating the prognostic ability of the 9 DEGs in the future. By then, the expression of these 9 DEGs may help predicting prognosis and overall survival time for patients with GC in future clinical practice.

There are still some limitations in this study. First of all, data samples are mainly from public databases, which are limited and single. We will further explore this in multi-center or multi-data sets. Then, this study had no underlying experimental and clinical validation. Therefore, basic experiments on DEGs associated with gut microbiota in GC will be further carried out in the future, mainly focusing on relevant mechanisms and signaling pathways.

In conclusion, we found that the 9 DEGs related to gut microflora, including *HSD17B3*, *GNG7*, *CHAD*, *ARHGAP8*, *NOX1*, *YY2*, *GOLGA8A*, *DNASE1L3*, and *ABCA8*, might be associated to prognosis of GC. Both risk signature and nomogram constructed using the 9 feature DEGs could effectively predict the prognosis for GC patients.

## Figures and Tables

**Table 1.** Distribution and Comparison of Clinical Parameters in Different Risk Groups

Characteristics	Total Cases (n = 91)	Group		*P*
		Low Risk (n = 45)	High Risk (n = 46)	
Age (years)				
≤60	30	13	17	5.05E-01
>60	61	32	29	
Gender				
Male	59	27	32	3.85E-01
Female	32	18	14	
Pathologic M				
M0	80	40	40	9.99E-01
M1	11	5	6	
Pathologic N				
N0	32	18	14	6.23E-01
N1	29	13	16	
N2	14	8	6	
N3	13	5	8	
Pathologic T				
T1	3	2	1	5.13E-01
T2	36	17	19	
T3	35	16	19	
T4	13	9	4	
Pathologic stage				
I	17	11	6	4.21E-01
II	32	14	18	
III	22	12	10	
IV	13	5	8	
Neoplasm histologic grade				
G1	3	2	1	1.79E-01
G2	31	19	12	
G3	57	24	33	
Microsatellite status				
MSS	55	32	23	1.86E-01
MSI-L	20	7	13	
MSI-H	15	6	9	
Recurrence				
Yes	21	5	16	1.82E-03
No	49	32	17	

**Table 2.** Biological Processes and KEGG Signaling Pathways Significantly Correlated with DEGs in the Correlation Network

Category	Count	*P*
Biology process		
GO:0006260~DNA replication	11	2.18E-06
GO:0070268~cornification	9	1.65E-05
GO:0031424~keratinization	10	1.78E-04
GO:0007052~mitotic spindle organization	8	2.57E-04
GO:0045109~intermediate filament organization	5	5.22E-04
GO:0007059~chromosome segregation	6	8.78E-04
GO:0007018~microtubule-based movement	6	1.71E-03
GO:0042572~retinol metabolic process	5	1.72E-03
GO:0006281~DNA repair	10	1.77E-03
GO:0010628~positive regulation of gene expression	14	1.90E-03
GO:0000278~mitotic cell cycle	7	3.10E-03
GO:0007568~aging	7	1.11E-02
GO:0030855~epithelial cell differentiation	5	1.31E-02
GO:0030198~extracellular matrix organization	8	1.49E-02
GO:0008284~positive regulation of cell proliferation	12	1.79E-02
GO:0006468~protein phosphorylation	11	2.87E-02
GO:0045892~negative regulation of transcription, DNA-templated	12	2.94E-02
GO:0051301~cell division	9	3.59E-02
GO:0008285~negative regulation of cell proliferation	10	3.81E-02
GO:0051897~positive regulation of protein kinase B signaling	6	4.36E-02
KEGG pathway		
hsa00830:Retinol metabolism	5	4.63E-03
hsa03440:Homologous recombination	4	7.78E-03
hsa05150:*Staphylococcus aureus* infection	5	1.53E-02
hsa04110:Cell cycle	5	3.69E-02
hsa00051:Fructose and mannose metabolism	3	4.26E-02
hsa04512: extracellular matrix-receptor interaction	4	4.58E-02
hsa04151:PI3K-Akt signaling pathway	8	4.63E-02
hsa04915:Estrogen signaling pathway	5	4.89E-02

DEGs, differentially expressed genes; GO BP, gene ontology biology process; KEGG, Kyoto Encyclopedia of Genes and Genomes.

**Table 3.** Clinical Characteristics of the Patients

Clinical Characteristics	UCSC (n = 91)	Univariable Cox			Multivariable Cox		
		HR	95%CI	*P*	HR	95%CI	*P*
Age (years, mean ± SD)	65.98 ± 11.49	1.005	0.976-1.035	7.45E-01	–	–	–
Gender (male/female)	59/32	0.862	0.449-1.656	6.55E-01	–	–	–
Pathologic M(M0/M1/-)	80/11	3.429	1.401-8.396	4.12E-03	3.067	1.014-12.83	4.750E-02
Pathologic N(N0/N1/N2/N3/-)	32/29/14/13/3	1.326	0.981-1.793	7.09E-02	–	–	–
Pathologic T(T1/T2/T3/T4/-)	3/36/35/13/4	1.194	0.764-1.867	4.35E-01	–	–	–
Pathologic stage (I/II/III/IV/-)	17/32/22/13/7	1.716	1.166-2.525	5.45E-03	1.401	0.829-2.370	2.083E-01
Neoplasm histologic grade (G1/G2/G3)	3/31/57	1.113	0.6178-2.005	7.22E-01	–	–	–
Microsatellite status (MSS/MSI-L/MSI-H)	55/20/15/1	1.167	0.799-1.704	4.22E-01	–	–	–
Recurrence (Yes/No/-)	21/49/21	1.973	0.925-4.208	7.34E-02	–	–	–
RS model status (high/low)	45/46	8.314	3.654-18.92	7.04E-09	10.959	4.291-27.99	5.610E-07

CI, confidence interval; HR, hazard ratio; RS, risk score; UCSC, University of California Santa Cruz.
